# The prevalence of documented cardiovascular-related pregnancy complications: cross-sectional study in an academic primary care centre

**DOI:** 10.3399/BJGPO.2022.0070

**Published:** 2022-11-30

**Authors:** Shivani Bhat, Debbie Elman, Aakriti Pyakurel, Karen Fleming

**Affiliations:** 1 Primary Care Research Unit, Department of Family and Community Medicine, Sunnybrook Health Sciences Centre, Toronto, Canada; 2 Specialised Foundation Programme, NHS Brighton and Sussex University Hospitals, Brighton, UK; 3 Academic Family Health Team, Sunnybrook Health Sciences Centre, Toronto, Canada

**Keywords:** cardiovascular pregnancy complications, cross-sectional studies, electronic health records, family practice, hypertension, pregnancy-induced, pregnancy, primary prevention, postpartum period, women’s health

## Abstract

**Background:**

Pregnancy and the postpartum period offer a unique opportunity to identify patients with risk factors leading to premature cardiovascular disease (CVD), which often go unrecognised.

**Aim:**

This study investigates self-reported prevalence of CVD-related pregnancy complications and its documentation in electronic medical records (EMRs) in an academic family health team (AFHT).

**Design & setting:**

A retrospective cross-sectional survey conducted from 2016 to 2017 in an AFHT.

**Method:**

The survey assessed self-reported pregnancy complications and obstetric histories of adult females. EMRs of responders who provided consent were appraised for documented pregnancy complications, and management of traditional cardiovascular risk factors post-pregnancy.

**Results:**

Out of 211 responders, 28% (*n* = 60) had at least one pregnancy complication reported in the survey and/or in the EMR, of which 67% (*n* = 40) had the complication documented in their EMR. The most prevalent complications were preterm birth (PTB; 12%, *n* = 25), hypertensive disorders of pregnancy (HDP; 10%, *n* = 22), and gestational diabetes mellitus (GDM; 7%, *n* = 14). Twenty-nine per cent (*n* = 4) of the patients with GDM had a 75 g oral glucose tolerance test result documented post-pregnancy. Of those with HDP, 36% (*n* = 8) had body mass index and 50% (*n* = 11) had a blood pressure measurement recorded after delivery.

**Conclusion:**

There has been a significant lack of documentation of pregnancy-related cardiovascular risk factors and subsequent management, introducing a missed opportunity for early cardiovascular intervention. Adequate documentation of pregnancy complications in the EMR and better transitions in care between obstetric and primary care teams could potentially enable clinicians to intervene early and better manage females at increased risk of CVD.

## How this fits in

There is robust evidence of the importance of capturing and following up on cardiovascular-related pregnancy complications. This study uniquely highlights the utility of EMRs in documenting cardiovascular-related pregnancy complications, allowing for better clinical follow-up. This study also reveals the lack of information flow and communication across obstetric and family health teams. The postpartum period provides a timely opportunity for family health teams to intervene and monitor maternal CVD risk factors that could potentially lead to early CVD; an opportunity that is easily missed in current practice.

## Introduction

CVD is the leading cause of death in females in Canada.^1^ Despite robust evidence highlighting the different cardiovascular risk factors between the sexes, its recognition in the clinical care of females has been slow or absent.^2,3^ In particular, pregnancy-related factors such as HDP, GDM, and PTB have all been established as independent risk factors associated with accelerated atherosclerosis and heart failure.^4–6^ The increased cardiometabolic demands of pregnancy may bring out early abnormalities that would otherwise remain silent. With the recent inclusion of sex-specific risk factors in Canadian clinical guidelines, clinicians are better equipped to integrate risk stratification, targeted screening, and follow-up management for sex-related cardiovascular risk factors into routine practice.^7–13^

Many females in Canada regularly access the healthcare system during pregnancy and childbirth. Therefore, pregnancy and the postpartum period provide a unique opportunity for GPs to intervene.^14^ However, with <10% of births being attended by GPs,^15^ they are often unaware of their patients’ pregnancy history.^16^ A 2015 survey of 504 randomly selected Canadian physicians also revealed substantial gaps in their knowledge regarding the prevalence and identification of heart disease among females. One-third of the physicians reported that more males than females die from CVD each year, whereas the prevalence is roughly equal, and only 39% of GPs were aware that pre-eclampsia doubles the risk of CVD after pregnancy.^17^ It is evident that along with gaps in practice, there are substantial deficiencies in knowledge regarding female-specific risk factors for CVD.

To tackle both gaps in knowledge and practice, EMRs have become the primary tool for physicians to document, monitor, and manage patients.^18^ With most GPs having access to EMRs in Ontario,^19^ electronic tools can be used to prompt physicians to ask patients about their medical and obstetric history, and to ensure appropriate screening and management of cardiovascular risk factors. According to the 2014 National Physician Survey, 65% of doctors said patient care improved after the implementation of EMRs owing to the ease and improved availability of lab results.^20^ EMRs have been shown to reduce the number of duplicate tests ordered, reduce adverse drug events and time spent on administrative tasks, as well as to improve both disease management and preventative care.^21,22^ While these electronic systems are being implemented in Ontario, they are often unique to one practice with lack of integration across healthcare systems.^23^

This study sought to: a) determine the prevalence of self-reported pregnancy-related complications in patients of an AFHT; b) assess the current state of EMR documentation of pregnancy complications; and c) assess whether follow-up is conducted in patients with pregnancy-related cardiovascular risk factors in an AFHT.

## Method

This study consisted of: a) a retrospective survey completed by patients who identified as female and were rostered to the AFHT; and b) a review of participant EMRs for documentation and assessment of pregnancy-related cardiovascular risk factors.

### Setting

This study was conducted in an AFHT situated in a primarily affluent neighbourhood in Toronto, Canada. This AFHT consists of 13 family physicians with varying practices in family medicine obstetrics (for example, some physicians provide antepartum care, some deliver babies at the hospital, some provide postpartum care only), and a large cohort of family medicine residents and nurses. Obstetrical care within the AFHT is provided by family doctors and family medicine residents, and care is shared with obstetricians if needed. Patients may have been cared for by obstetricians, midwives, or family physicians outside the AFHT. Patients of the AFHT have access to a wide array of dedicated interprofessional health providers including diabetes teams, dietitians, and nurse practitioners, in addition to the specialist and diagnostic services of the hospital.

### Participants

All female patients registered with the AFHT were initially contacted by letter and email, if available. Each patient was given a unique identifier number at the initial point of contact to match the responses to their pregnancy data. The letter gave patients the option to complete an online survey or an enclosed paper survey returned in a prepaid envelope. Responses were collected from December 2016 to March 2017. Owing to the limited availability of the population, a convenience sample was used. Participants were eligible if they identified as female, were aged 18–50 years, and had been pregnant and delivered at least once in their life.

### Survey

Maternal recall of obstetrical history have been seen to be an accurate characterisation of past pregnancy complications.^24^ For this study, a maternal recall questionnaire was designed to assess obstetrical and postpartum history. It included questions on sociodemographics, obstetric care providers (OCPs: GP, midwife, or obstetrician), obstetric history including pregnancy complications, medical history, level of physical activity, and information on postpartum visit including whether the patient recalls having a conversation about their obstetric history with their physician. The survey is included in Supplementary Box S1.

EMRs of all participants who provided written consent in the survey were accessed and reviewed, regardless of whether they self-reported a pregnancy complication in the survey. Data were extracted using the automated search feature and manual extraction (specified in Supplementary Box S2). Specifically, the cumulative patient profile, consultation notes in text, and attached PDFs were searched for in the EMR used by the family practice. To ensure comprehensive audit, the central hospital EMR was searched to obtain any missing data on the cardiometabolic profile and laboratory results. Pregnancy complications were considered as ‘documented’ if they were explicitly written in any aspect of the EMR. Each patient ID was multiplied by a random factor to generate de-identified IDs for analysis. The principal investigator and statistician were both blinded to the patient key.

For the current study, GDM was defined by clinical criteria or ‘gestational diabetes’ written in the patient’s chart by a clinical provider. Pre-eclampsia was defined by new onset hypertension (systolic blood pressure ‡140 mmHg or diastolic blood pressure ‡90 mmHg) and proteinuria (>300 mg in 24 hours or a protein-to-creatinine ratio >0.20) after a gestational age of 20 weeks. PTB was defined as delivery before a gestational age of 37 weeks. Postpartum follow-up data extracted from the EMR included 75 g oral glucose tolerance test (OGTT) measured 6 weeks to 6 months postpartum, and maternal blood pressure and body mass index measured 6 weeks to 6 months postpartum.

### Data analysis

Descriptive statistics were calculated to describe the characteristics of the participants, prevalence of pregnancy complications in the sample, and follow-up data. Although many of the data for the purposes of this study’s analysis were complete, any missing data were managed via pairwise deletion. Data were analysed using MS Excel and SAS (version 9.4). All electronic survey responses are stored on the OceanStudies server (an encrypted database on a Canadian data server) and all paper surveys are stored securely at the hospital’s research unit for up to 7 years.

## Results

**Figure 1.**
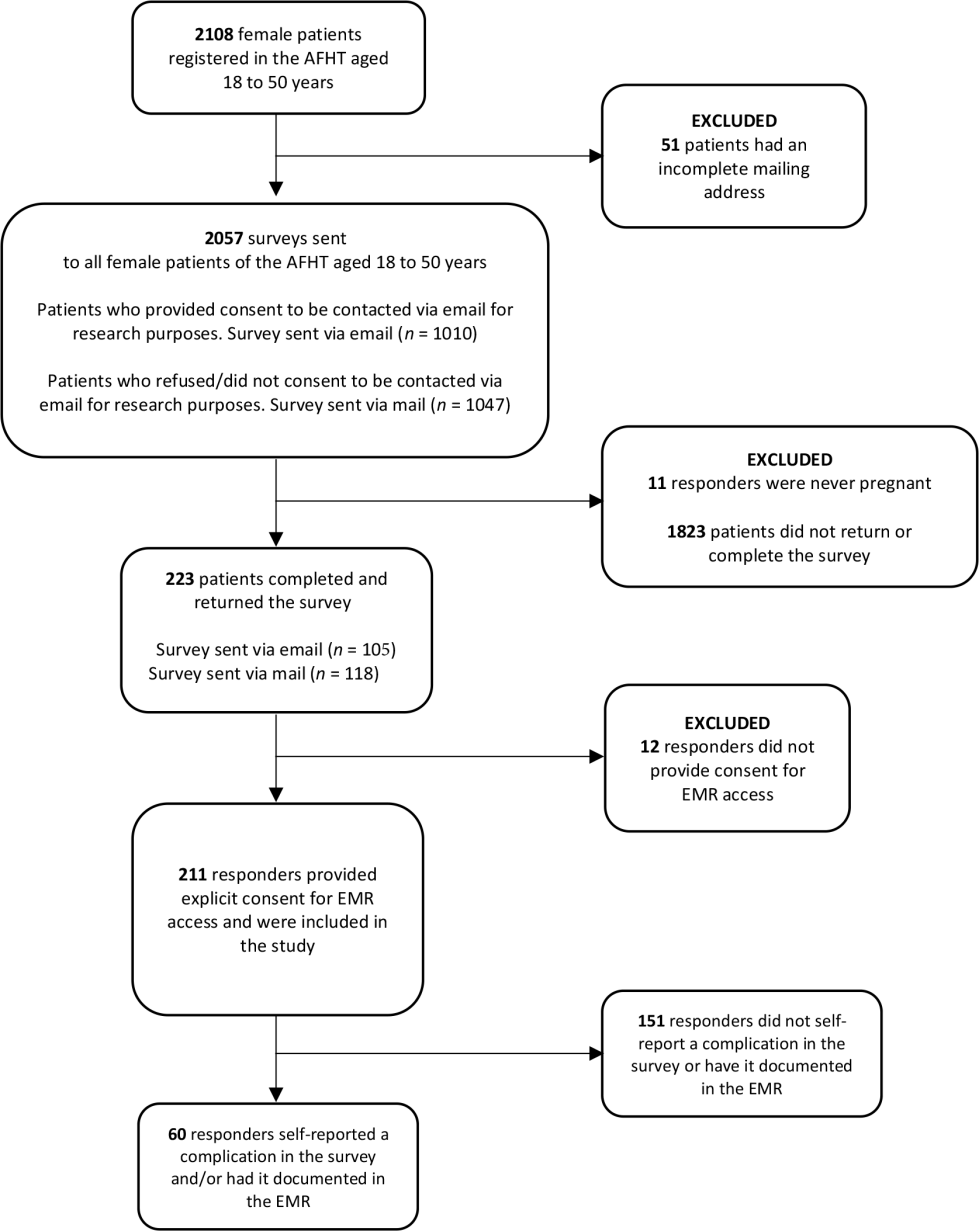
Study participants flowchart. AFHT = academic family health team. EMR = electronic medical record.

With 234 responses (response rate of 11%), 11 (5%) had never been pregnant, resulting in 223 (95%) eligible participants. Of these responders, 211 (95%) provided consent to access their EMR (Figure 1).

### Patient characteristics

In general, responders were middle-aged, affluent, white, married with a post-secondary qualification, and reported excellent health status, closely representing the profile of the community served by the AFHT (see Supplementary Box S3). Overall, 137 (65%) responders recalled discussing their obstetric history with their family doctor, of which 61 (45%) were cared for by their regular family doctor during their pregnancy.

### Pregnancy complications

**Figure 2.**
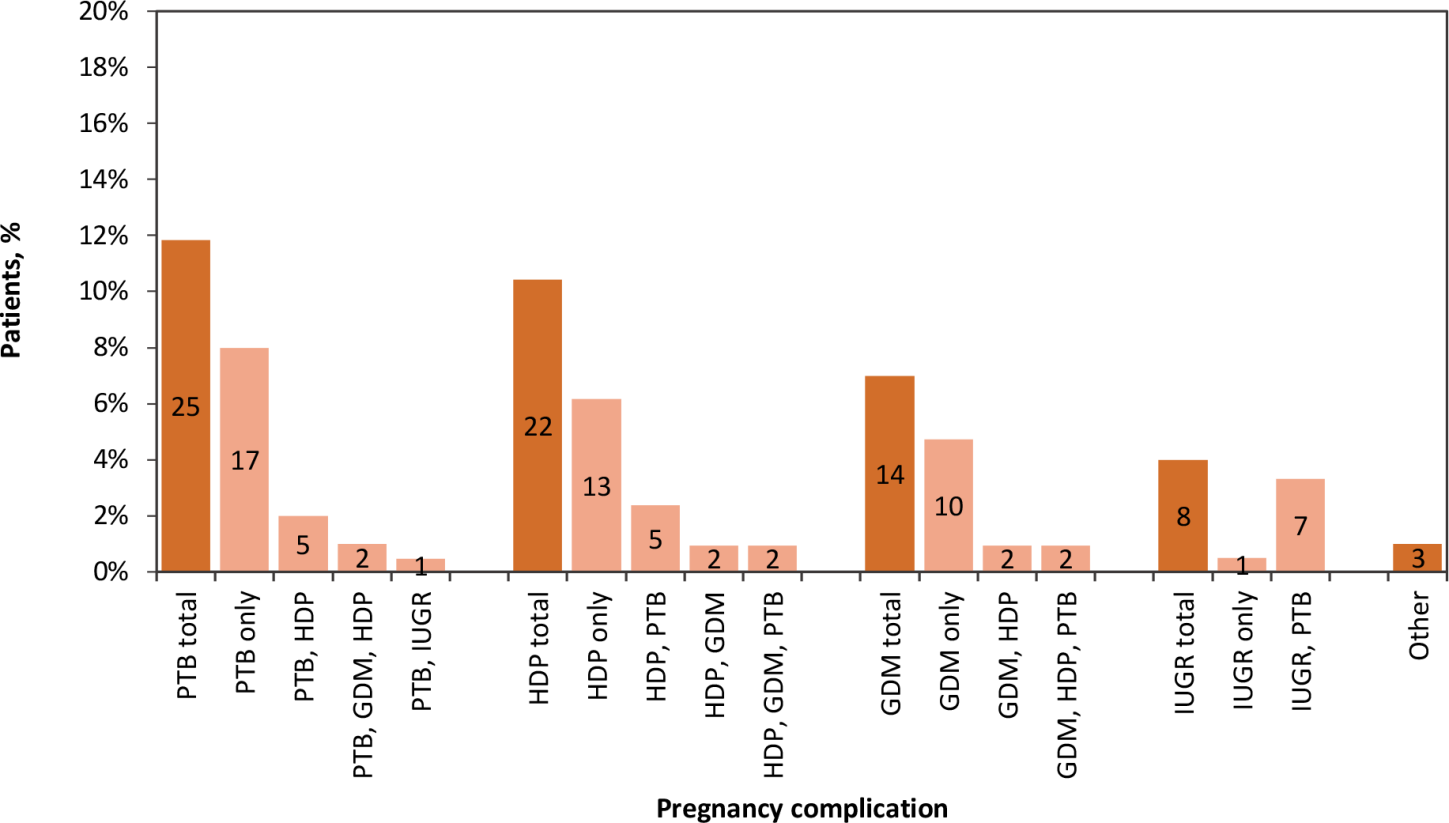
All pregnancy complications reported in survey and/or listed in the EMR of patients who consented to chart review (*n* = 211). Number of patients specified as labels. GDM = gestational diabetes mellitus. HDP = hypertensive disorders of pregnancy. IUGR = intrauterine growth restriction. PTB = preterm birth.

**Figure 3.**
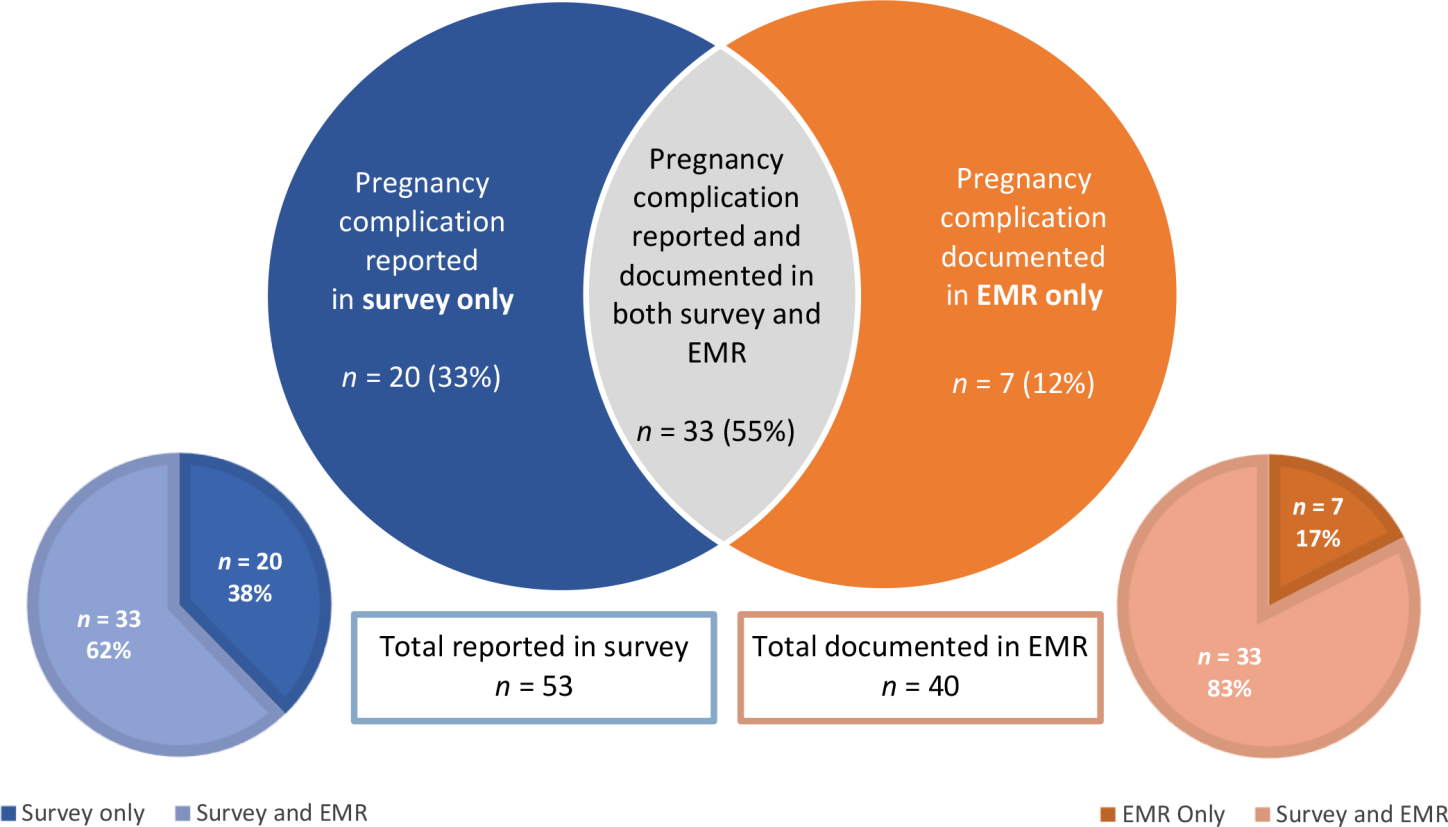
Diagram indicating whether the pregnancy complication was reported in the survey and/or EMR (electronic medical record) of all responders who had a pregnancy complication and consented to chart review (*n* = 60).

**Figure 4.**
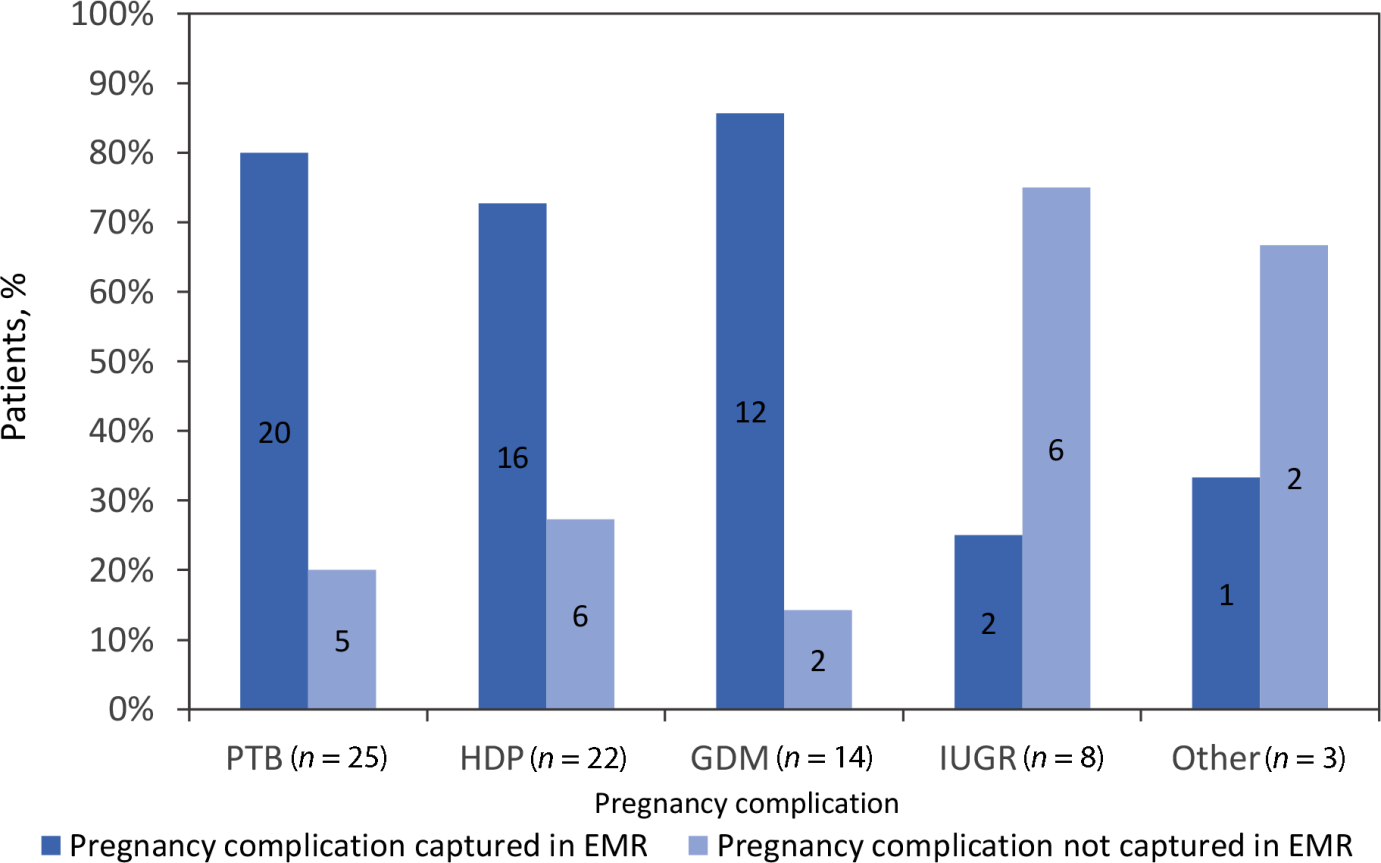
Documentation of pregnancy complications according to type of pregnancy complication. GDM = gestational diabetes mellitus. HDP = hypertensive disorders of pregnancy. IUGR = intrauterine growth restriction. PTB = preterm birth.

Of the 211 participants who consented to chart review, 60 (28%) had at least one complication self-reported in the survey and/or documented in the EMR (Figure 2). In summary, 40 (67%) responders with a complication had it recorded in their EMR, regardless of their response (Figure 3). The complications most documented in the EMR were PTB, HDP, and GDM, as illustrated in Figure 4.

The odds of documentation of pregnancy complications in patients cared for by their primary family doctor during pregnancy were not statistically significant (odds ratio 0.74; 95% confidence interval = 0.23 to 2.32) (data not shown).

### Follow-up of risk factors

Only 29% of responders who experienced GDM had a postpartum 75 g OGTT recorded in their chart. Of those who experienced HDP during pregnancy, 36% had a body mass index and 50% had a blood pressure measurement recorded in their EMR post-pregnancy (Table 1).

**Table 1.** Postpartum follow-up of essential risk factors documented in the EMR

Description	Recorded in EMR postpartum, *n* (%)
**GDM**	14
75g OGTT result	4 (29)
**HDP**	22
Blood pressure measurement	11 (50)
Recent SBP ≥130mmHg	6 (27)
BMI	8 (36)

BMI = body mass index. EMR = electronic medical record. GDM = gestational diabetes mellitus. HDP = hypertensive disorders of pregnancy. OGTT = oral glucose tolerance test. SBP = systolic blood pressure.

## Discussion

### Summary

This study offers initial insights into the lack of documentation of pregnancy complications in EMRs, revealing a missed opportunity to identify and manage females at increased risk of CVD. EMRs function as an efficient tool to facilitate documentation of medical histories, which is the first step in enabling clinicians to better manage patient outcomes. Improvement of documentation of pregnancy complications in the EMR has the potential to ensure greater physician awareness of patient history and bridge transitions in care.^18,25^

Owing to opportunities for shared obstetric care in AFHT, several healthcare providers other than the GP could be involved in a patient’s intrapartum and postpartum care, which could potentially lead to increased gaps in communication and information flow. Several scenarios in shared care could lead to differences in documentation of pregnancy complications and communication. Patients may have been cared for by their primary family doctor with care transferred to an obstetrical provider before the complication occurring. Without detailed discharge summaries, documentation within the EMR or direct communication between the obstetrician and family physician, there are few possibilities to truly understand which of these scenarios each of the shared care patients falls under, thereby introducing numerous possibilities in the lack of documentation and follow-up.^26^

### Strengths and limitations

This study has several limitations and, in particular, has implications on generalisability to other clinical and geographic settings. The participants in this sample were predominantly white, highly educated with adequate financial stability each month, and had access to a Diabetes Education Centre, a breastfeeding clinic, and an AFHT in a large tertiary care hospital. They likely had higher levels of health literacy and self-advocacy than the general public. Furthermore, 36% of them received obstetric care from their regular family doctor (see Supplementary Box S3), facilitating ready access to information about complications. This is a higher rate than seen provincially or nationally.^27^ Even within this high socioeconomic community with access to several tertiary care services, there was a notable gap in documentation of pregnancy complications and, consequently, follow-up. Therefore, these findings would possibly be worse in settings with ethnic minorities and low socioeconomic strata. This inference further emphasises the need to find solutions to bridge the lacunae in documentation and communication of pregnancy complications.

Maternal recall surveys have been shown to have high specificity and negative predictive value, indicating that self-reporting of pregnancy complications experienced 3–6 years ago is likely to be accurate. Aspects of pregnancy such as birth weight, PTB, and pre-eclampsia have been seen to be accurately reported by females decades after a pregnancy.^28^ Despite that, there is still an element of recall bias, since some of the pregnancies in this study dated back >10 years. There may also have been self-selection bias in completing the survey resulting in the low response rate and the small sample size. Although the prevalence of pregnancy complications in this study is mostly similar to national figures, a larger sample size would provide opportunity for regression analyses, where correlations between OCP and pregnancy complication follow-up could be further investigated.

Differences in EMR systems between the family practice and hospital departments is probably one of most notable limitations in ensuring adequate postpartum follow-up. In Canada, a patchwork of EMR systems has developed as a result of decentralised administration of health care from the federal government to individual provinces, and from the provinces to the local level. Due to a lack of computer literacy, low compatibility with other systems, time constraints, and inadequate clinician involvement, the adoption of EMR across the nation has suffered.^29^ These barriers are seen at the provincial level as well.^21^ In Ontario, EMRs are mostly used in family practices, as is the case for the AFHT in this study.^18^ However, owing to differences in software used between the family practice and the hospital, successful interdepartmental communication is inadequate, resulting in missed opportunities of chronic disease prevention as evident in this study.

### Comparison with existing literature

This study highlights several gaps in communication and clinical practice as identified in previous studies. In a survey assessing GP knowledge, attitudes, and practices regarding pregnancy complications, only 50% of family physicians reported collecting pregnancy history from their patients.^30^ Similarly, only 62% of the participants in thissample who self-reported a pregnancy complication in the survey had it recorded in their EMR. The lack of communication between OCPs and GPs likely plays a major role in suboptimal documentation of these patients. MacDonald *et al^16^* reported that 83% of maternity care providers in Ontario claimed they inform GPs about the patient’s pregnancy complication, notably HDP, which is incongruous with the 58% of the GPs who reported they were informed of the same. There is a clear discrepancy between what the GPs believe they are told and what the OCPs believe they are informing.^16^ Lack of information flow is amplified because of the absence of standardised transfer of care protocols that include pregnancy complications. This is contrary to, for example, protocols in midwifery care.^31^ As a result, GPs often rely on their patient and/or OCP to relay pregnancy-related information, which may not be sustainable or efficient for patient care.

Similar to national figures, responders had a 7% and 10% overall prevalence of GDM and HDP, respectively. GDM was reasonably well captured, which may be because of the early introduction of GDM management guidelines in 2008 and a greater proportion of studies conducted on diabetes and GDM.^32^ Although GDM was well documented, postpartum 75 g OGTT was suboptimal, despite access to comprehensive programmes in self-management of diabetes via Diabetes Education Centres,^33^ revealing a gap in practice.

HDP was not as well documented as GDM, where only 50% of the participants had their blood pressure documented within 6 months of delivery. HDP has only been established as an independent risk factor for CVD in the last decade.^14,34,35^ While the Society of Obstetricians and Gynaecologists of Canada (SOGC) has postpartum guidelines for monitoring patients with HDP, there is no guideline specific to primary care, which is where much of the postpartum cardiovascular risk management occurs.^34,35^ GPs may lack awareness of SOGC guidelines, possibly owing to their target audience being obstetrical providers and gynaecologists.^17^ Since HDP was first recently added as a cardiovascular risk factor by the Canadian Cardiovascular Society in 2016 and then by Hypertension Canada in 2018,^11,12^ the uptake of the clinical guidelines by GPs may potentially be evident in the coming years.

Interestingly, the prevalence of PTB in this sample (12%) was higher than national figures (8%).^36^ On further examination, one-third had a PTB pregnancy complicated by intrauterine growth restriction (IUGR), HDP, and GDM— all of which are established factors associated with PTB.^37^ Furthermore, of the responders who experienced PTB owing to factors other than HDP, GDM, and IUGR (*n* = 8), seven (88%) were aged 30–39 years and one (13%) was a current/previous smoker (data not shown), factors that have been associated with an increased risk of PTB.^38^ Finally, the small sample number is likely playing an important role. Because of heterogeneous factors associated with PTB,^39^ its prevalence may not accurately predict the isolated burden of the syndrome in this sample.

### Implications for research and practice

Pregnancy offers a unique window to a woman’s future health. The link between pregnancy complications and future CVD provides physicians with perhaps the earliest opportunity for disease prevention. GPs deal with preventive medicine and motivational interviewing on a daily basis and are therefore well positioned to identify and counsel patients at increased risk of chronic disease. This study reinforced the need for a multifaceted approach to improve documentation and management of obstetric complications to improve future cardiovascular health. In addition to systemic changes that would facilitate or mandate sharing of information between care providers, there is a need for improved knowledge translation in primary care as well as tools to improve documentation and follow-up.

After this study, the team initiated several strategies to address some of these gaps. Patient education postcards were developed that encouraged patients to share their pregnancy history with their family doctor. Additionally, a well-designed e-Module series on HDP is in development for family medicine residents; currently, these topics receive scant attention within postgraduate curricula. Finally, the research team is leading an initiative to design and develop an EMR-based tool that facilitates information gathering, documentation, and management of pregnancy complications. Through multidisciplinary collaborations, it seeks to enable females and their physicians to improve postpartum cardiovascular risk management and to achieve improved long-term health goals.

## Data

This dataset is not publicly available.
